# Strong sub-terahertz surface waves generated on a metal wire by high-intensity laser pulses

**DOI:** 10.1038/srep08268

**Published:** 2015-02-05

**Authors:** Shigeki Tokita, Shuji Sakabe, Takeshi Nagashima, Masaki Hashida, Shunsuke Inoue

**Affiliations:** 1 Advanced Research Center for Beam Science, Institute for Chemical Research, Kyoto University, Gokasho, Uji, Kyoto 611-0011, Japan; 2 Department of Physics, Graduate School of Science, Kyoto University, Kitashirakawa, Sakyo, Kyoto 606-7501, Japan; 3 Institute of Laser Engineering, Osaka University, 2-6 Yamada-oka, Suita, Osaka 565-0871, Japan

## Abstract

Terahertz pulses trapped as surface waves on a wire waveguide can be flexibly transmitted and focused to sub-wavelength dimensions by using, for example, a tapered tip. This is particularly useful for applications that require high-field pulses. However, the generation of strong terahertz surface waves on a wire waveguide remains a challenge. Here, ultrafast field propagation along a metal wire driven by a femtosecond laser pulse with an intensity of 10^18^ W/cm^2^ is characterized by femtosecond electron deflectometry. From experimental and numerical results, we conclude that the field propagating at the speed of light is a half-cycle transverse-magnetic surface wave excited on the wire and a considerable portion of the kinetic energy of laser-produced fast electrons can be transferred to the sub-surface wave. The peak electric field strength of the surface wave and the pulse duration are estimated to be 200 MV/m and 7 ps, respectively.

Ultrashort terahertz pulses are now utilized not only as probe pulses, but also as ultrafast driving pulses in materials science and photonics experiments^1^,^2^,^3^,^4^,^5^,^6^. Such investigations rely on picosecond or sub-picosecond terahertz pulses with field strengths greater than 10 MV/m. Terahertz sources utilizing laser plasmas are a promising technology for creating table-top equipment to generate extremely strong terahertz pulses with energies approaching the millijoule level, because laser plasmas would overcome the limitations associated with damage in nonlinear crystal-based terahertz sources^7^,^8^,^9^,^10^,^11^. For example, efficient terahertz pulse generation has recently been demonstrated by irradiating a thin metal foil with a laser pulse having an intensity of 5 × 10^19^ W/cm^2^; a pulse energy of ≥460 μJ and a conversion efficiency of ≥0.07% was achieved by collecting free-space radiation with a large divergence angle using a ellipsoidal mirror^11^. In addition, terahertz pulses trapped as surface waves (surface plasmon polaritons) on a metal wire can be flexibly transmitted^12^ and focused to sub-wavelength dimensions by techniques such as using a tapered tip^13^,^14^. This is particularly useful for applications that require high-field pulses. However, the generation of strong terahertz surface waves (for example, with a field strength of the order of 100 MV/m) remains a challenge due to the large coupling loss between free-space waves and surface waves for broadband ultrashort terahertz pulses^15^,^16^,^17^.

The processes of energy conversion and transport through the relativistic interactions of high-intensity laser pulses (*I* > 10^18^ W/cm^2^) with solid targets have been widely investigated for their potential applications, such as ion acceleration^18^, fast ignition fusion^19^, and terahertz wave generation. Some remarkable phenomena have also been observed in relativistic laser interactions with wire targets^20^,^21^,^22^,^23^,^24^,^25^. Field propagation at the speed of light along a wire has been observed by laser-driven proton radiography^22^ and is attributed to a transient current with a peak magnitude of the order of 10^4^ A. The collimation and guidance of fast electrons with energies of a few hundred keV has been demonstrated using long wire targets^23^,^24^, suggesting that a strong transient field is generated on the wire. However, in such phenomena involving large energy transport over long distances along the target surface, the dynamics of the fields and fast electrons are not fully understood.

High-time-resolution measurement of the transient electric and magnetic fields accompanying the generation and transport of fast electrons is a powerful means of investigating such phenomena. Radiography using laser-driven proton beams has been widely utilized^22^,^25^,^26^, but the time resolution depends on the pulse duration of the laser-accelerated proton beam and is constrained to a few picoseconds at best. Utilizing the coulomb interaction of laser-driven electron pulses, we have recently measured the emission duration of fast electrons with sub-picosecond resolution at a laser intensity of 10^16^ W/cm^2^^27^. Field measurement using ultrashort electron pulses holds considerable promise for the high-time-resolution observation of plasma phenomena because femtosecond electron pulses accurately synchronized with laser pulses can be generated by laser acceleration.

In this paper, we report high-time-resolution measurements of the temporal waveform of an electromagnetic field, induced by a femtosecond laser pulse with an intensity of 10^18^ W/cm^2^, propagating along the surface of a metal wire. The measurement is realized by a new technique for ultrafast field measurement based on femtosecond electron deflectometry using laser-accelerated and recompressed electron pulses. We observe a strong pulse signal propagating at the speed of light, even though there is no significant number of high-energy fast electrons propagating at that speed. Experimental results show that the pulse signal is due to a surface wave rather than fast electrons. Numerical simulations indicate that a sub-terahertz transverse-magnetic (TM) surface wave was efficiently excited by laser-plasma interaction in the metal wire targets.

## Results

**Figure 1:**
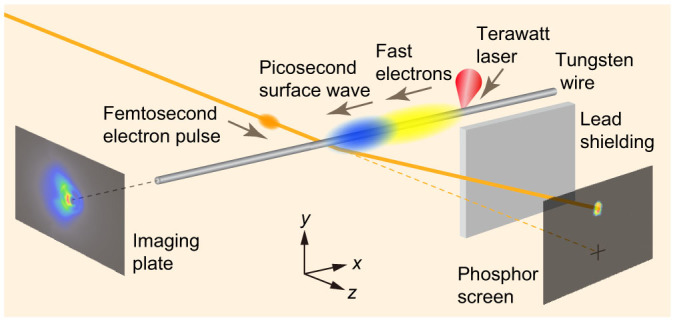
Experimental layout for femtosecond electron deflectometry measurement and emission distribution measurement of fast electrons. The wire is moved after each laser irradiation so that a fresh part of the wire surface is used. A lead plate is placed to shield the phosphor screen from fast electrons and X-rays emitted from the laser-irradiated spot. Fast electrons emitted from the laser-irradiated spot in a direction along the wire are detected by stacked imaging plates.

A schematic of the experimental setup is shown in Fig. 1. Laser pulses are provided by a 150 fs Ti: sapphire laser system. A *p*-polarized laser pulse with an energy of 70 mJ is focused onto one side of a 0.3 mm diameter tungsten wire at an incidence angle of 55°. The focused beam has a waist of 6 μm × 4 μm in full width at half-maximum (FWHM), resulting in a peak intensity of 1 × 10^18^ W/cm^2^. For probing the electric and magnetic fields, a 500 fs electron pulse with an energy of 390 keV is generated by an apparatus for laser acceleration and pulse compression^28^; the probe pulse is passed near the wire and detected on a phosphor screen with a charge-coupled device camera placed 85 mm from the wire. The distance between the center of the wire and the electron beam axis is *y_d_* = 0.5 mm. The origin of the time delay (coincidence of arrival time at the wire of the laser pulse and the probe electron pulse) is determined by a method based on ponderomotive scattering^29^; the error in determining the delay is at most ±0.5 ps. The experiments are performed in a vacuum chamber with a pressure of 0.1 Pa.

**Figure 2:**
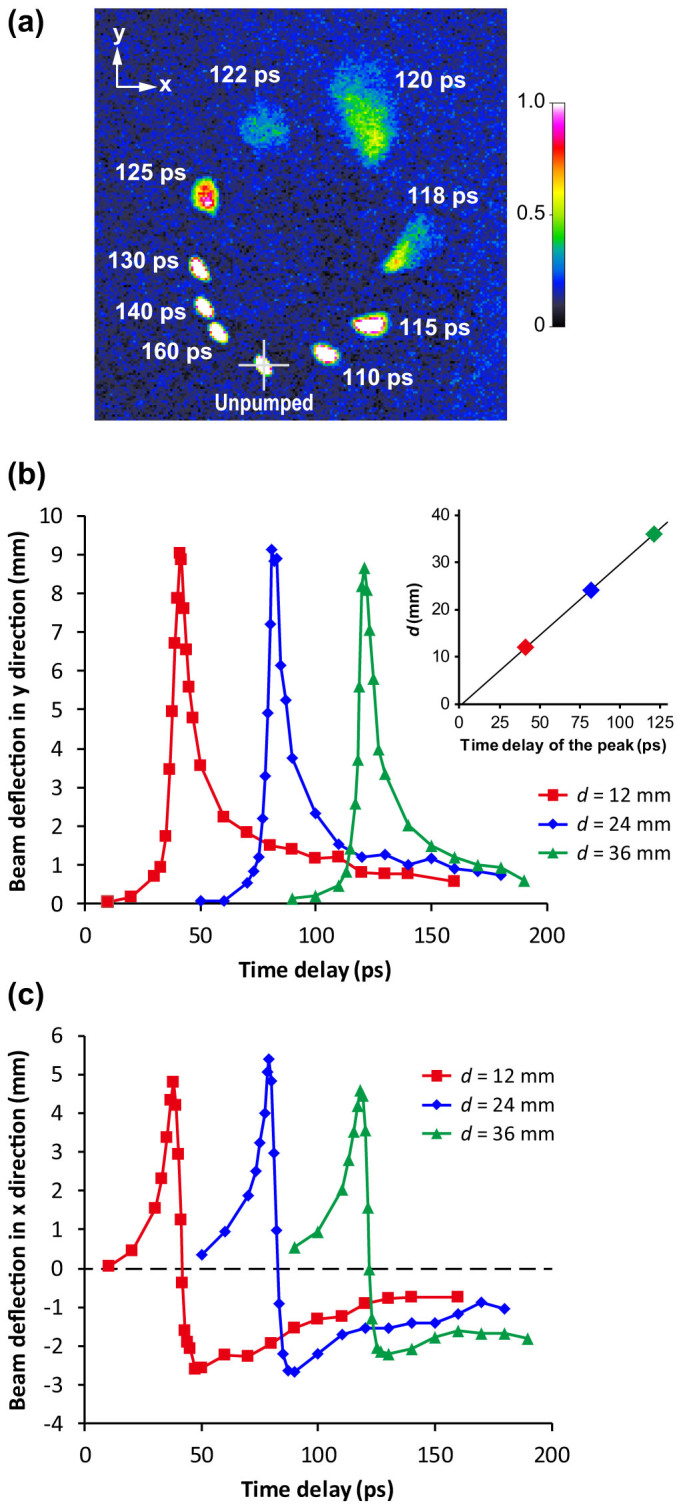
Surface waveform measurement by electron beam deflectometry. (a) Electron beam images detected on the phosphor screen at *d* = 36 mm. This is a composite image of 10 single-shot images for different delay durations. (b) Time traces of the beam deflection in the *y* direction. Inset shows a plot of *d* versus the time delay of the peak of the time trace. (c) Time traces of the beam deflection in the *x* direction.

Electron beam deflection is measured by varying the delay between the laser and the electron pulse at *d* = 12 mm, 24 mm, and 36 mm, where *d* is the distance from the laser-irradiated spot to the position where the electron beam crosses the wire. The distance from the end of the wire to the position where the probe beam crosses the wire is about 28 mm. Figure 2(a) shows the electron beam image for each delay. The electron beam is deflected in both the *y* direction (perpendicular to the wire) and the *x* direction (parallel to the wire), and a trace of the beam on the screen describes an oval. Figures 2(b) and 2(c) show the time traces of beam deflection in the *y* and *x* directions, respectively. Each time trace of the *y* deflection has nearly the same shape and contains a single peak with FWHM of approximately 10 ps. The velocity at which the peak moves along the wire is measured to be (0.99 ± 0.01)*c*, where *c* is the speed of light.

Let us consider two possible explanations for these results. One is that relativistic electrons guided along the wire^23^,^24^ are detected. In this case, the fast electrons should be highly relativistic (kinetic energy *K* = *m_e_c*^2^[(1−*v*^2^/c^2^)^−1/2^−1] > 2 MeV, where *m_e_* and *v* are, respectively, the rest mass and velocity of the electron) because the propagation velocity is very close to the speed of light (*v* > 0.98*c*). The other is that an electromagnetic surface wave is excited on the wire. In this case, a significant fraction of the surface wave power should be reflected at the end of the wire due to the impedance mismatch between free space and the surface wave mode, and this reflection can be detected. To decide between these two possibilities, we performed two experiments.

**Figure 3:**
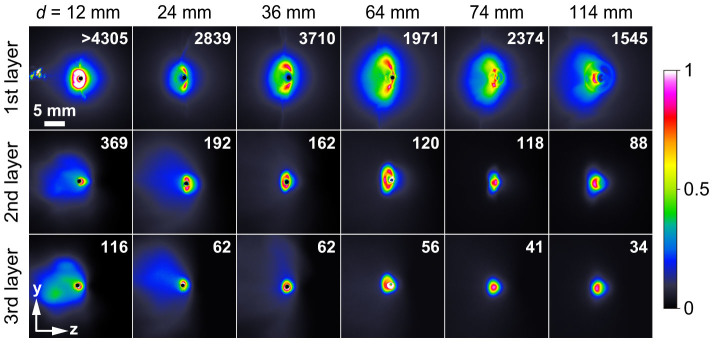
Fast electron images detected by stacked imaging plates. The images are taken for a single shot at *d* = 12 to 114 mm. The distance from the laser-irradiated spot to the end of the wire is 64 mm. The imaging plates used for *d* = 12 to 64 mm have a pinhole of 1 mm in diameter to allow the wire to pass through. The value indicated in each images is the integrated count of the electron signal. The color scale is set independently for each image, according to the indicated contrast scale factor, to provide maximum contrast.

First, the fast electrons emitted from the laser-irradiated spot in a direction along the wire (negative *x*-direction) were detected by triple-layer stacked imaging plates (IPs) at distances *d* = 12 to 134 mm, as shown in Fig. 3. The imaging plates are highly sensitive to electrons in the energy range from 40 to 1000 keV, with a sensitivity peak at around 200 keV. The second and third layers of the stacked imaging plates detect electrons with energies higher than about 400 and 600 keV, respectively. The electron distributions beyond the end of the wire (*d* > 64 mm) are very similar to those reported previously^23^. The total charge of the electrons detected at the first layer IP is estimated to be 1.5–3 nC and 1–1.5 nC for *d* ≤ 64 mm and *d* > 64 mm, respectively. The integrated counts of electron signals in the second layer IP are more than one order of magnitude lower than in the first layer IP, so it is estimated that more than 90% of the electrons have an energy lower than 400 keV. Thus, it is clear that the majority of the fast electrons have velocities substantially slower than the speed of light.

**Figure 4:**
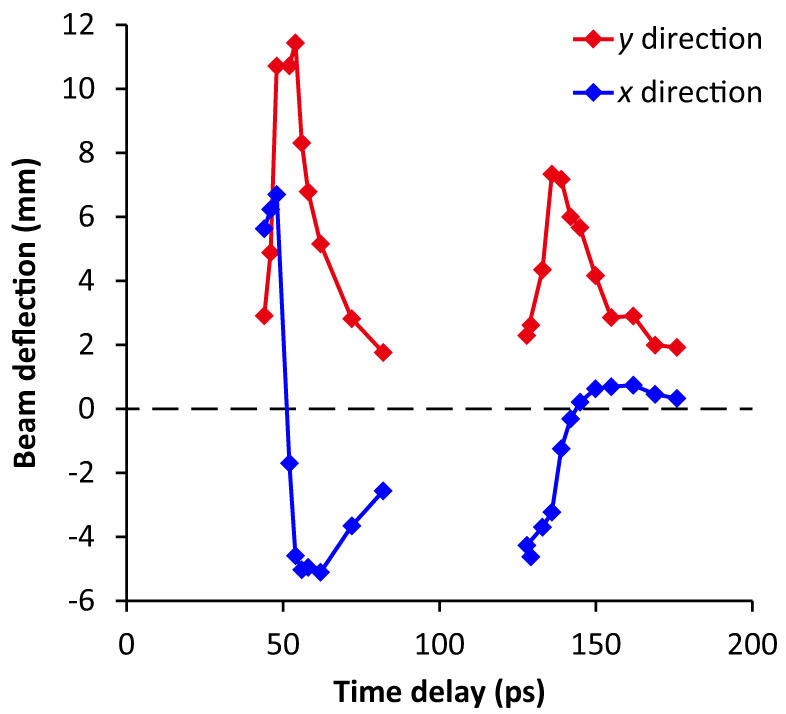
Time traces of electron beam deflections in the *x* and *y* directions for the waveform measurement of a reflected surface wave at *d* = 15 mm. The distance from the end of the wire to the position where the probe beam crosses the wire is 13 ± 0.5 mm.

Second, to observe the reflected pulse by using the electron probe within the adjustable delay range, the distance between the end of the wire and the position where the probe beam crosses the wire was shortened from 28 mm to 13 mm. The results are shown in Fig. 4. A peak at 51 ps and another at 137 ps are observed in the time trace of the *y* deflection. The delay of *Δt* = 86 ps between the two peaks agrees with the round-trip time of a pulse propagating at the speed of light over the distance between the detection point and the end of the wire (*c*·*Δt* ≈ 2 × 13 mm). As described below, the *y* and *x* deflections correspond to the electric and magnetic field strengths, respectively, when assuming a fundamental TM surface wave. Thus, it is consistent that the time traces of the *x* deflections around 51 and 137 ps have opposite slopes, because the electromagnetic wave should have opposite magnetic fields before and after reflection as a consequence of traveling in opposite directions. Taken together, these results strongly suggest that a strong surface wave propagates along the metal wire.

In the gigahertz to terahertz frequency range, a fundamental TM wave can be guided as a surface wave along a single metal wire; this is an example of the well-known Sommerfeld wave^30^. Sommerfeld waves have a radially polarized electric field, an azimuthally polarized magnetic field (with a transverse field distribution approximated by 1/*r*, where *r* is the radial distance from the wire), and a propagation speed extremely close to the speed of light. The longitudinal component of the electric field can be ignored because it is 2–3 orders of magnitude smaller than the transverse component. We can therefore treat the *y* and *x* beam deflections separately as effects caused by the electric and magnetic fields, respectively. The beam deflection angle as a function of the delay *τ* can be approximated as a convolution of the temporal waveform of the surface wave and the Lorentz force profile around the wire: 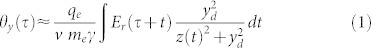

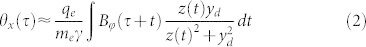


Here, *q_e_* and *v* are, respectively, the charge and velocity of the electron, and *γ* is the Lorentz factor. Therefore, if the waveforms of *E_r_* and *B_φ_* are Gaussian, the trace of the electron beam on the screen (*x*-*y* plane) describes an oval. Using these relations, we estimate the pulse duration (FWHM) of the electric and magnetic field to be 7 ps from measurements of the time traces shown in Figs. 2(b) and 2(c), assuming a Gaussian pulse shape. We also estimate the peak strengths of the electric and magnetic fields at the surface of the wire to be 198 MV/m and 0.66 T, respectively, for *d* = 12 mm. The ratio of the electric and magnetic field is *E_r_*/*H_φ_* ≈ 377 which is good agreement with those for Sommerfeld waves (*E_r_*/*H_φ_* ≈ 376.8 at 0.1 THz). Regarding the characteristics of the generated surface wave, we can roughly estimate that its frequency range is from 0 to 0.1 THz (center frequency of 0 Hz), the peak electric current induced in the wire is 500 A, the peak power is greater than 70 MW, and the pulse energy of the main peak is greater than 400 μJ. Here, the pulse energy is estimated from the volume integral of the Poynting vector by assuming the theoretical field distribution (given by a Hankel function) of the Sommerfeld wave^30^.

**Figure 5:**
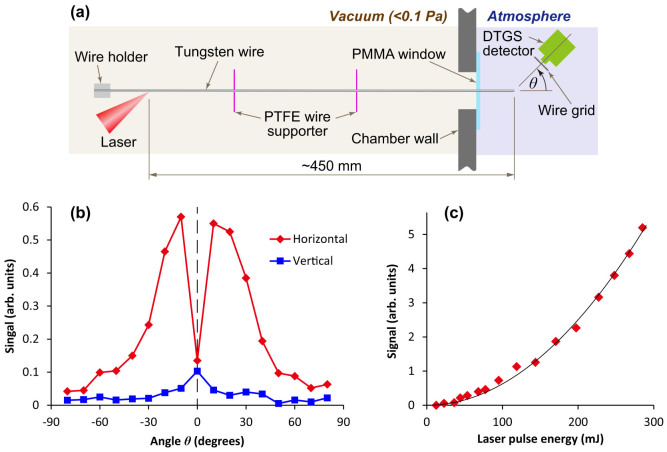
Detection of electromagnetic waves emitted from the end of a wire of 500 mm in length, using a deuterated triglycine sulfate (DTGS) pyroelectric detector. (a) Experimental layout for radiation detection. (PTFE: polytetrafluoroethylene. PMMA: Poly(methyl methacrylate).) The distance from the laser-irradiated spot to the end of the wire is about 450 mm, and the distance from the end of the wire to the pyroelectric detector is 50 mm. A black polypropylene sheet is put over the detector window to block scattered light. (b) Angular distribution of radiation with horizontal and vertical polarizations in the horizontal plane. The incident laser pulse energy is 80 mJ. (c) Dependence of signal on laser pule energy at 20° for horizontal polarization. The solid line is a parabola (*y* = *a*·*x*^2^) fitted to the experimental data, which are indicated by the diamond symbols.

Since the propagation loss of the wire waveguide in the gigahertz to terahertz frequency range is low (e.g., 2 dB/m at 100 GHz (theoretical value)), the generated surface wave can be transmitted over a long wire. As shown in Fig. 5(a), if a tungsten wire of 500 mm in length is irradiated with 150 fs laser pulses in a vacuum, then the generated surface wave can be guided into the atmosphere via a wire transmission line spanning a distance of about 450 mm. The surface wave is reflected at the end of the wire, but there will be partial emission of an electromagnetic wave into free space. The strength and polarization of the emitted wave is detected by using a deuterated triglycine sulfate (DTGS) pyroelectric detector with a terahertz wire-grid polarizer. The measured angular distributions of radiation with horizontal and vertical polarizations in the horizontal plane are shown in Fig. 5(b). The signals of the vertically polarized components are comparable to the noise level, while the signals of the horizontally polarized components are strong between 10° and 40° and between −10° and −40°. In contrast, in the vertical plane, strong vertical signals and weak horizontal signals were observed in additional experiments. These results indicate that the detected radiation has a radial polarization originating from the Sommerfeld surface wave. Figure 5(c) shows the dependence of the signal on laser pulse energy at 20° for the horizontally polarized component. The signal intensity is proportional to the square of laser pulse energy and the signal does not saturate at the maximum laser pulse energy of 285 mJ. From the ratio of the signals at 70 and 285 mJ, we estimate that the total energy of the surface wave reaches approximately 5 mJ at the maximum laser pulse energy, resulting a conversion efficiency of approximately 1.7%.

**Figure 6:**
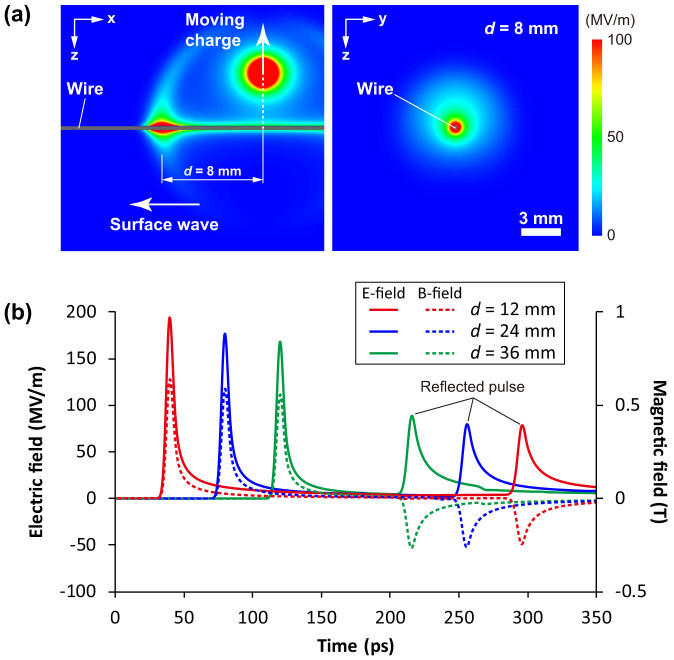
Results of numerical simulation of the electromagnetic field. The diameter of the tungsten wire is 0.3 mm. The length of wire between the position of electric charge emission and the end of the wire is 50 mm. (a) Snapshots of the distribution of the absolute electric field in cross sections along and across the wire axis. The *x*, *y*, and *z* coordinates correspond to those in Fig. 1. (b) Temporal waveforms of the radial electric field (solid lines) and azimuthal magnetic field (dashed lines) on the wire surface at the positions *d* = 12, 24, and 32 mm.

Numerical simulations are necessary to understand the mechanism of the surface wave generation. However, two- and three-dimensional particle-in-cell codes, which are the most common methods used in laser-plasma simulations, have not generally been used for such a large target (a few centimeters) because of computational limits. Here, using a finite-difference time-domain algorithm, we perform a three-dimensional simulation of surface wave generation and propagation with a simplified electric current that approximates the emission of fast electrons from the laser-spot. Typically, fast electrons generated by laser-plasma interactions with solids at the irradiation spot are emitted in almost all directions^31^, and have a wide energy distribution^32^. The average and total kinetic energies of the fast electrons strongly depend on the interaction mechanism, but are generally of the order of a hundred keV^33^ and ten to several tens of percent of the laser pulse energy^31^, respectively, for a laser intensity of 10^18^ W/cm^2^. It should be noted that the fast electron pulse emitted into the vacuum from the laser-spot has a short temporal duration but it expands rapidly due to wide velocity and angular distributions. Therefore, the pulse duration of an electromagnetic wave excited by the fast electron current could be longer than the laser pulse duration. However, the contribution to the surface wave excitation from the fast electrons at places far from the wire surface should be small, because the electric field distribution of a Sommerfeld wave is localized close to the wire surface. Taking these factors into account, we adopt a very simple model for a surface wave excitation which is driven by the radial component of the fast electron current flowing toward the vacuum from the surface of a wire target. We assume a negative charge moving with a constant velocity of 0.5*c* (corresponding to the velocity of a 79 keV electron). We neglect the electromagnetic forces, and the spatial charge density is Gaussian with a FWHM of 0.45 mm (3 ps in duration) in the direction of propagation. Figure 6(a) shows a snapshot of the calculated electric field distribution: the propagating electric field surrounding the wire is radially polarized and is nearly axisymmetric. The simulation, therefore, indicates that a Sommerfeld surface wave is excited by the moving electric charge. When we assume an amount of electric charge of 16 nC, the peak electric and magnetic fields at the surface of the wire reach 194 MV/m and 0.64 T, respectively. The temporal waveforms of the radial electric field and azimuthal magnetic field at positions *d* = 12, 24, and 32 mm are illustrated in Fig. 6(b), which show that the waveforms have a main peak and a long tail. The pulse duration of the main peak is 6.7 ps (FWHM), but that of the reflected pulse has increased to 11.2 ps. Also, the peak intensity of the reflected pulse is about half of the initial value. These simulation results are consistent with the experimental results. The total kinetic energy of the 16 nC moving electric charge is about 1.3 mJ, which corresponds to 2% of the laser pulse energy in our experiment. This ratio is reasonable in terms of energy balance, because it is lower than the typical laser-to-fast-electron energy conversion efficiency at a laser intensity of the order of 10^18^ W/cm^2^^31^. The total energy of the surface wave in the simulation is calculated to be 410 μJ, which corresponds to 32% of the total kinetic energy of the moving charge, but this ratio might be overestimated because the energy loss of the moving charge is neglected. From the simulation, we conclude that a fast electron current in the radial direction can couple efficiently to a picosecond half-cycle surface wave. Moreover, additional simulations show that the pulse energy of the surface wave increases in proportion to the square of the amount of electron charge. The laser-to-surface-wave energy conversion efficiency is, therefore, expected to rise with increasing laser pulse energy, which is consistent with experimental results shown in Fig. 5(c).

## Discussion

We have reported the observation of a strong surface wave produced by relativistic laser interaction with a metal wire. We have also demonstrated that femtosecond electron deflectometry using laser-accelerated electrons can be used as a powerful method for time- and space-resolved quantitative measurement of ultrafast electromagnetic dynamics induced by laser-plasma interactions in the relativistic intensity regime. From our experimental and numerical results, we find a considerable portion of the kinetic energy of fast electrons can be transferred to a low-frequency surface wave. The energy transfer to surface waves in ultrafast laser-plasma interactions not only cannot be ignored as an energy loss mechanism for fast electrons, but also can significantly influence the dynamics of the emission of electrons and ions. In fact, the electric and magnetic fields of the surface wave are strong enough to affect the trajectories of fast electrons with energies of the order of MeV. We, therefore, suggest that the mechanism of collimated fast electron emission along a wire^23^,^24^ is related to the surface wave. Moreover, we expect that effects of the reflection and interference of surface waves in laser-plasma interactions will be observed in the future.

From the viewpoint of potential applications, metal wires can serve as excellent low loss and low dispersion waveguides for Sommerfeld waves in the gigahertz to terahertz frequency range^12^,^30^,^34^. The strong surface waves generated on a metal wire can therefore be efficiently transmitted from the laser-produced hot plasma to a cold laboratory environment. Although the efficiency of the energy conversion from the laser to the surface wave was estimated to be around 1%, we believe there is further room for improvement by, for example, simply increasing the laser power. This convenient and power-scalable terahertz source opens new avenues for experimental investigations in plasma and beam physics, terahertz photonics, and materials science. In power-scaling, however, an ultimate limit for surface wave intensity exists, which is attributed to ionization of the wire surface by a propagating surface wave. Experimental investigation of the limits of surface wave intensity in wire waveguides might be possible using existing high power lasers with powers up to a petawatt.

## Methods

### Femtosecond electron pulse generation and detection

Probe electron pulses were generated by relativistic intensity laser pulses incident on a polyethylene film^28^. The two laser pulses (one for surface wave generation and one for probe pulse generation) were obtained by splitting a single laser beam. The temporal delay between the two laser pulses was adjusted by an optical delay line using a hollow retroreflector. The laser pulse for probe generation had a pulse energy of 80 mJ and a focused intensity of 1 × 10^18^ W/cm^2^. Fast electrons emitted from the film were collimated to a low-divergence beam of 1 mm in diameter by using an aperture and a permanent magnetic lens. Then, the electron pulse was guided into a pulse compressor composed of two permanent dipole magnets with a beam bending angle of 180°. In the field created by the dipole magnets, higher energy electrons followed a longer path than those with lower energy. A slit was placed at the energy-dispersive plane between the two dipole magnets in order to select the electron energy. The momentum spread of the selected electrons was estimated to be 1%. After passing through the pulse compressor, the electron pulse had a 500 fs duration and was focused by a magnetic lens system to a spot of 100 μm in diameter. The pulse duration was measured by a method based on ponderomotive scattering^29^. The origin of the delay (coincidence of arrival time at the wire of the laser pulse and the probe electron pulse) was also determined by the same method; the error in determining the delay was within ±0.5 ps. The energy of the electron pulse, determined by using an electron diffraction pattern from a gold crystal, was 390 ± 10 keV. A phosphor screen and a lens-coupled electron-multiplying CCD camera were used for probe beam measurement. The total number of electrons in the probe pulse which were detected at the phosphor screen was estimated to be of the order of 3 × 10^4^.

## Author Contributions

S.T. is the principal investigator who proposed and performed the experiment and the numerical simulation. S.S. is a coordinator of the experiment and the leader of the project. T.N. prepared the pyroelectric detector and gave advice on the measurements. M.H. and S.I. supported the preparation and implementation of the experiment. All authors reviewed the manuscript.
